# Perception of primary care doctors towards telemedicine in Kuching, Sarawak: A cross-sectional study

**DOI:** 10.51866/oa.505

**Published:** 2024-02-19

**Authors:** Xu Vin Su, Sutiman Isnani, Wan Mohamad Suut Sharifah Khadijah, Hoklai Sarudu Shareezan, Lih Kai Lau, Ishak Maziah, Nur Amani Ahmad Tajuddin, Chun Wai Chan

**Affiliations:** 1 MD, Klinik Kesihatan Petra Jaya, Jalan Siol, Kanan, Kuching, Sarawak, Malaysia. Email: xuvinz88@gmail.com; 2 MD, Klinik Kesihatan Petra Jaya, Jalan Siol, Kanan, Kuching, Sarawak, Malaysia.; 3 MD, Klinik Kesihatan Petra Jaya, Jalan Siol Kanan, Kuching, Sarawak, Malaysia.; 4 MD, Klinik Kesihatan Batu Kawa, Jalan Stapok Utara, Taman Stapok, Kuching, Sarawak, Malaysia.; 5 MD, Klinik Kesihatan Batu Kawa, Jalan Stapok Utara, Taman Stapok, Kuching, Sarawak, Malaysia.; 6 MD, MFamMed, Klinik Kesihatan Petra Jaya, Jalan Siol, Kanan, Kuching, Sarawak, Malaysia.; 7 MBBS, MFamMed, Department of Primary Care Medicine, Faculty of Medicine, Universiti Malaya, Kuala Lumpur, Malaysia.; 8 MD, MPH, DABFM, Department of Family Medicine, International Medical University, Jalan Rasah, Seremban, Negeri, Sembilan, Malaysia.

**Keywords:** Telemedicine, Primary health care, Perception

## Abstract

**Introduction:**

Telemedicine is the provision of healthcare remotely via information and communications technology (ICT). This study aimed to assess the familiarity and factors related to the perception towards telemedicine and the willingness to practise telemedicine among primary care doctors.

**Methods:**

A multi-centre cross-sectional study was conducted prospectively at all public healthcare clinics across Kuching, Sarawak. A questionnaire was adapted and modified from an overseas validated questionnaire, consisting of four parts: demographic data, familiarity towards telemedicine, factors related to the perception of telemedicine and willingness to implement telemedicine.

**Results:**

A total of 131 doctors were recruited. Of them, 43.5% had never interacted with patients via email, WhatsApp or Telegram, while 68.7% had never attended any conferences, speeches or meetings regarding telemedicine. The doctors had low familiarity towards guidelines, technology and medical applications of telemedicine. The majority agreed on the ability of telemedicine to save patients’ time and money, the potential of ICT in healthcare and the necessity during a pandemic but perceived the possibility of technical difficulties. The doctors who had experience in interacting with patients via email, WhatsApp or Telegram (P=0.001) and those who had ≤8 years of working experience (P=0.04) had a significantly better perception towards telemedicine.

**Conclusion:**

Although the familiarity towards telemedicine among public primary care doctors is low, their perception is good in a majority of areas. Adequate technological support and continuous education on telemedicine and its guidelines, especially medicolegal issues, are imperative to adopt and propagate telemedicine in primary care.

## Introduction

Telemedicine is defined as the provision of healthcare remotely via information and communications technology (ICT).^1^ It includes store-and-forward, remote patient monitoring and real-time interactive services.^2^ Globally, the adoption of telemedicine has escalated tremendously since the COVID-19 pandemic. Although conditions vary between countries, there is a positive role of telemedicine in improving health system performances and outcomes in both developed and developing countries.^3^ The telemedicine global market was valued at 50 billion US dollars in 2019 and is projected to be valued at nearly 460 billion US dollars by 2030.^4^ Telemedicine use has increased 38 times since the pre-COVID-19 period owing to strong continued uptake, positive consumer perception and tangible investment in this area.^5^

In Malaysia, telemedicine was initially introduced via hospital information systems, aiding the healthcare cluster concept (lead hospitals sharing services with non-lead hospitals).^6^ It then further expanded throughout the country mostly involving private tertiary care centres and primary care providers, including the use of DoctorOnCall, Speedoc and HomeGP for remote consultations. The expansion and increased consumer uptake of telemedicine in Malaysia are in line with Malaysia’s Telemedicine Blueprint established by the Ministry of Health (MOH) on 25 July 1997, which envisioned healthcare system transformation and advancement in ICT by 2020.^7^ Telemedicine in Malaysia is regulated by the Malaysian Medical Council Advisory on Virtual Consultation (during the COVID-19 pandemic) under the Medical Act 1971 (amended in 2012).^8^ The MOH Malaysia started the implementation of the Virtual Clinic programme at five clinics in government primary care settings in August 2019. The programme expanded to 230 clinics in 2022, with the target of expanding to 100 additional clinics in 2023.^9^

In the past, telemedicine had been typically used in specialised care; currently, its utilisation for routine patient care is being recognised. Although the benefits of telemedicine are vast, challenges in implementing telemedicine arise, including cost issues, technical difficulties, medicolegal issues, patient education, patients’ willingness to participate in telemedicine services and quality of care. This study aimed to assess the awareness and factors related to the perception of telemedicine among primary care doctors to enable stakeholders to strategise and have a targeted approach in improving the literacy, acceptance and widespread implementation of telemedicine throughout the state and country. It also aimed to evaluate the willingness of primary care doctors to practise telemedicine.

## Methods

A multi-centre cross-sectional study was conducted prospectively at all public healthcare clinics across Kuching, Sarawak. The study received ethical approval from the Medical Research and Ethics Committee, MOH, Malaysia. A self-administered questionnaire was employed via Google Forms to collect data from July to October 2022. The questionnaire was adapted and modified from an overseas validated questionnaire, with permission obtained from the original authors.^10^ Content validation was conducted via a questionnaire content review by five experts. These experts consisted of family medicine specialists at primary care clinics implementing the Virtual Clinic programme. Face validation was performed by obtaining feedback from 10 primary care doctors. A pilot study was conducted among 15 participants. Universal sampling was applied at all public healthcare clinics across Kuching. Of a total population of 148 doctors, 131 were recruited, yielding a response rate of 88.5%. All participants were briefed using an information sheet and provided consent on Google Forms by clicking on a box stating their agreement.

The questionnaire consisted of four parts: demographic data, familiarity towards telemedicine, factors related to the perception of telemedicine and willingness to implement telemedicine. The questions for familiarity were scored using a 5-point Likert scale: 1=very low, 2=low, 3=average, 4=high and 5=very high. Similarly, the questions for perception and willingness were scored using a scale: 1=strongly disagree, 2=disagree, 3=neutral, 4=agree and 5=strongly agree.

Both descriptive and inferential statistical analyses were performed using IBM SPSS version 27.^11^ The mean value of the dependent variables was interpreted based on the interval shown in Table 1.^12^ The total score for the familiarity and perception towards telemedicine was obtained by summing the scores in the Likert scale for each domain and converting the scores to be over 100 for the inferential data analysis. Analysis of Variance (ANOVA) was applied to evaluate the association of the sociodemographic characteristics with the familiarity and perception towards telemedicine and willingness to practise telemedicine. Pearson correlation coefficients were used to test the correlation of the sociodemographic characteristics with the familiarity and perception towards telemedicine and willingness to practise telemedicine as well as the correlation between each domain. Multivariate analysis was conducted with a general linear model for variables with a P-value of <0.25. Statistical significance was accepted at a confidence level of 95% and a P-value of <0.05.

**Table 1:** Five-point Likert scale for the mean scores for the familiarity and perception of the doctors towards telemedicine.

Likert scale	Interval	Difference	Description of familiarity	Description of perception and willingness
1	1.00-1.79	0.79	Very low	Strongly disagree
2	1.80-2.59	0.79	Low	Disagree
3	2.60-3.39	0.79	Average	Neutral
4	3.40-4.19	0.79	High	Agree
5	4.20-5.00	0.80	Very high	Strongly agree

## Results

The mean age of the doctors was 33.93 years. The majority of the doctors were women (67.9%), were medical officers (89.3%) and had a working experience of <8 years (61%). Table 2 shows the sociodemographic characteristics and computer literacy and access of the doctors. These data showed a normal distribution.

**Table 2:** Descriptive analysis of the independent variables.

Variables	Subset	% (n)	Mean	SD
Age	>33 years ≤33 years	42.0 (55) 58.0 (76)	33.93	4.91
Sex	Male Female	32.1 (42) 67.9 (89)		
Professional ranking	House officer Medical officer Specialist	6.9 (9) 89.3 (117) 3.85 (5)		
Number of years of service	>8 years ≤8 years	38.9 (51) 61.0 (80)	8.45	4.69
Average number of patients consulted in a day	≥40 <40	50.3 (66) 49.6 (65)	36.09	15.71
Hours per day of PC/laptop usage at home	>1 hour ≤1 hour	55.0 (72) 45.0 (59)	2.42	2.38
Hours per day searching for information online	>1 hour ≤1 hour	61.1 (80) 38.9 (51)	2.82	2.58
Number of smart devices	>2 ≤2	38.9 (51) 61.0 (80)	2.34	1.00
Frequency of interaction with patients via email, WhatsApp or Telegram	0 hours/week <1 hour/week 1–2 hours/week 2–3 hours/week >3 hours/week	43.5 (57) 34.4 (45) 8.4 (11) 2.3 (3) 11.5 (15)		
Frequency of patients’ request to communicate via email, WhatsApp or Telegram	0 times/week <1 time/week 1–2 times/week 2–3 times/week >3 times/week	52.7 (69) 29.0 (38) 11.5 (15) 3.1 (4) 3.8 (5)		
Attendances to any conferences, speeches or meetings regarding telemedicine technology	0 1 2 3 >3	68.7 (90) 13.0 (17) 7.6 (10) 5.3 (7) 5.3 (7)		

Note - SD: Standard Deviation, PC: Personal Computer

Table 3 presents the mean scores for the familiarity and perceptions of the doctors towards telemedicine. The scores indicated that the doctors had low familiarity towards telemedicine. In particular, they had very low familiarity towards telemedicine guidelines (mean score=1.67). Nonetheless, the doctors exhibited a high degree of perception towards most factors related to telemedicine. Although the doctors remained neutral in their perception of whether telemedicine can replace physical clinic visits and whether telemedicine can save costs in healthcare services for the government (mean score=2.76 and 3.35, respectively), most of them agreed on telemedicine being necessary during a pandemic that hinders patients from attending clinic consultation (mean score=4.13) and on the potential of ICT in healthcare (mean score=4.05).

**Table 3:** Descriptive analysis of the dependent variables.

Factors	n	Mean	Description	SD	CI
**Familiarity towards telemedicine**					
Familiarity with telemedicine guidelines	131	1.67	Very low	0.80	[1.53, 1.81]
Familiarity with telemedicine technology (e.g. teleconferencing)	131	2.37	Low	1.05	[2.18, 2.55]
Familiarity with the medical applications of telemedicine technology	131	2.24	Low	0.96	[2.08, 2.41]
Total score	131	41.88		16.81	[39.0, 44.8]
**Perception towards telemedicine**					
There are few technical difficulties (IT equipment and infrastructure) in implementing telemedicine.	131	2.44	Disagree	1.09	[2.25, 2.63]
Telemedicine can replace physical clinic visits.	131	2.76	Neutral	1.19	[2.56, 2.97]
There are few concerning possible ethical and legal issues around interacting with patients online (e.g. patient confidentiality).	131	2.79	Neutral	1.09	[2.61, 2.98]
Telemedicine can save costs in healthcare services for the government.	131	3.35	Neutral	0.98	[3.18, 3.52]
Telemedicine systems can reduce workload.	131	3.61	Agree	1.00	[3.44, 3.78]
Telemedicine is an alternative to physician consultation with patients.	131	3.62	Agree	0.85	[3.47, 3.77]
Telemedicine systems can save healthcare workers’ time and money.	131	3.83	Agree	0.88	[3.68, 3.98]
Telemedicine systems can be integrated within the existing physical clinic system.	131	3.87	Agree	0.80	[3.73, 4.01]
Telemedicine is needed regardless of a pandemic.	131	3.88	Agree	0.88	[3.73, 4.03]
Telemedicine systems can save patients’ time and money.	131	3.92	Agree	0.80	[3.78, 4.06]
Potential of ICT/internet in healthcare	131	4.05	Agree	0.74	[3.93, 4.18]
Telemedicine is necessary during a pandemic that hinders patients from attending clinic consultation.	131	4.13	Agree	0.84	[3.99, 4.27]
Mean total score	131	70.43		12.08	[68.3, 72.5]
**Willingness to implement telemedicine**					
The implementation of telemedicine technology is appropriate owing to the current conditions in clinics.	131	3.49	Agree	0.91	[3.32, 3.65]
I think that my colleagues would be willing to implement telemedicine technology.	131	3.52	Agree	0.87	[3.37, 3.67]

Most participants agreed that the implementation of telemedicine technology is appropriate owing to the current conditions in clinics (mean score=3.49) and perceived their colleagues to be willing to implement telemedicine (mean score=3.52).

Table 4 illustrates the doctors’ preferences in telemedicine consultation. The majority of the doctors preferred to consult follow-up cases via telemedicine. The top three disciplines of cases preferred to be consulted via telemedicine were chronic non-communicable diseases, infectious diseases and psychiatric cases. Most doctors agreed that telemedicine is applicable in urban areas rather than in rural areas. The most preferred method of delivery of telemedicine was video calls.

**Table 4:** Doctors’ preferences in telemedicine consultation.

Preference in telemedicine consultation	% (n)
**Types of cases to consult**	
Follow-up cases	74.8 (98)
Both new cases and follow-up cases	25.2 (33)
New cases only	0 (0)
**Preferred discipline of cases to consult**	
Chronic non-communicable diseases (e.g. hypertension or diabetes mellitus)	94.6 (124)
Infectious diseases (e.g. tuberculosis, retroviral disease, rabies or hand–foot–mouth disease)	46.5 (61)
Psychiatric cases	34.3 (45)
Maternal health cases	16.0 (21)
Child health cases	11.4 (15)
Others (e.g. COVID-positive cases with comorbidities, dermatology cases or blood result review)	5(7)
**Area where telemedicine is applicable**	
Urban areas	90.8 (119)
Suburban areas	48.8 (64)
Rural areas	22.1 (29)
**Preferred method of telemedicine delivery**	
Video calls (e.g. Zoom, WhatsApp or Google Meet)	85.4 (112)
Telephone calls	57.2 (75)
Bookdoc, DoctorOnCall, Speedoc or Home GP	48.8 (64)
Messaging applications (e.g. WhatsApp, Telegram, WeChat or Facebook Messenger)	45.8 (60)
Emails	29.0 (38)

ANOVA showed a significant association of greater familiarity towards telemedicine with an average number of patients consulted in a day of >40 (F=4.24, P=0.041), >1 hour of PC/laptop usage in a day (F=8.04, P=0.005), more than two smart devices owned (F=8.82, P=0.004), higher frequency of interaction with patients via email, WhatsApp or Telegram (F=7.04, P=0.009), higher frequency of patients’ request to communicate via email, WhatsApp or Telegram (F=7.49, P=0.007) and attendance to one or more conferences, speeches or meetings regarding telemedicine technology (F=49.62, P<0.001). The multivariate analysis with the general linear model also demonstrated significance at P<0.05 in these variables, except for the number of hours of PC/laptop usage in a day, which could be a confounding factor (Table 5). No significance was found between the other sociodemographic characteristics and familiarity towards telemedicine.

**Table 5:** General linear model analysis of the scores for the familiarity and perception towards telemedicine.

Variables	n	Mean	Std error	CI	P
**Familiarity towards telemedicine**					
*Average number of patients seen in a day*					
<40	65	41.84	1.75	[38.39, 45.30]	0.003
≥40	66	48.95	1.81	[45.36, 52.54]	
*Number of smart devices*					
≤2	80	42.14	1.61	[38.95, 45.33]	0.008
>2	51	48.65	1.96	[44.77, 52.53]	
*Interaction with patients via email, WhatsApp or Telegram*					
No	57	42.64	1.92	[38.84, 46.44]	0.022
Yes	74	48.16	1.63	[44.92, 51.39]	
*Attendance to telemedicine conferences, speeches or meetings*					
0 times	90	36.65	1.46	[33.77, 39.53]	<0.001
≥1 times	41	54.14	2.20	[49.78, 58.50]	
**Perception towards telemedicine**					
*Number of years of service*					
≤8 years	80	71.67	1.30	[69.10, 74.23]	0.04
>8 years	51	67.39	1.61	[64.20, 70.57]	
*Interaction with patients via email, WhatsApp or Telegram*					
No	57	66.18	1.53	[63.16, 69.21]	0.001
Yes	74	72.87	1.36	[70.17, 75.56]	

Based on the Pearson correlation coefficients, a positive significant correlation was found between the familiarity towards telemedicine and the number of hours of PC/laptop usage in a day (r=0.31, P=<0.001), number of smart devices owned (r=0.21, P=0.015), frequency of interaction with patients via email, WhatsApp or Telegram (r=0.22, P=0.014), frequency of patients’ request to communicate via telemedicine modalities such as email, WhatsApp or Telegram (r=0.29, P=<0.001) and attendances to any conferences, speeches or meetings regarding telemedicine technology (r=0.52, P=<0.001).

A greater perception towards telemedicine was significantly associated with <8 years of service (F=4.79, P=0.03) and higher frequency of interaction with patients via email, WhatsApp or Telegram (F= 11.44, P=<0.001). This association remained significant in the multivariate analysis, although the magnitude between the covariances was not large (Table 5). No significance was found between the other sociodemographic factors and perception towards telemedicine.

The Pearson correlation coefficients revealed no significant correlation between the continuous independent variables and the perception of the doctors towards telemedicine. There was also no significant correlation found between the sociodemographic characteristics and doctors’ perception of the appropriateness to implement telemedicine in current clinic conditions and their colleagues’ willingness to implement telemedicine. Conversely, there were positive significant correlations between the doctors’ perception of the appropriateness to implement telemedicine technology at primary care clinics and familiarity towards telemedicine (r=0.19, P=0.031), perception towards telemedicine (r=0.42, P<0.001) and perception of their colleagues’ willingness to implement telemedicine (r=0.46, P<0.001).

## Discussion

### Interpretation of the main findings

In this study, 61.1% of the primary care doctors spent more than 1 hour per day searching for information online and 55.0% of them spent more than 1 hour on their PC/laptop at home. Further, 51% of primary care doctors owned more than two smart devices. A prior study reported that 91% of healthcare professionals used their smart devices in their workplaces.^13^ This finding reflects the high level of ICT exposure in a majority of doctors.

There was a lack of interaction with patients via email, WhatsApp or Telegram, as 77.9% of the primary care doctors in this study spent less than 1 hour per week for this purpose. This finding contradicts that of the study conducted by Albarrak et al. in Riyadh, where almost 72% of health practitioners had frequently interacted with patients via email or social media.^10^ This difference may be attributed to a lack of requests from patients for telemedicine services. In the present study, 81.7% of the doctors received requests from patients to communicate via telemedicine less than once a week. Another possible reason is the diflerence in the cultural and socioeconomic background and telemedicine exposure of patients between regions.

This study noted very low scores for the familiarity towards telemedicine guidelines and low scores for the familiarity towards telemedicine technology and medical application of such technology. Similarly, Albarrak et al. and Ayatollahi et al. found that clinicians’ knowledge of telemedicine technology was limited despite the common usage of telemedicine in many countries.^10,14^ Approximately 68.7% of the doctors in the current study had no prior exposure to conferences, speeches or meetings regarding telemedicine. Higher average number of patients seen in a day, higher number of smart devices owned, interaction with patients using telemedicine modalities and exposure or attendance to telemedicine conferences, speeches or meetings were significantly associated with greater familiarity towards telemedicine. These findings suggest that with more exposure to patients and telemedicine technology, doctors tend to gain more insights into telemedicine features, hence boosting their familiarity towards it.

The primary care doctors in this study perceived that there are technical difficulties in implementing telemedicine. Among technical barriers to telemedicine implementation are poor internet connection and a lack of universal access to technology.^15^ A lack of knowledge in telemedicine software navigation and language barrier are also challenges faced by patients. These challenges may be associated with racial, ethnic and socioeconomic differences between patients.^16^ The city of Kuching covers urban and suburban areas, with variation in internet coverage in diflerent areas.

In this study, the doctors were neutral about medicolegal concerns and the ability of telemedicine to replace physical clinic visits. These data reflect the possibility of the doctors’ lack of knowledge about medicolegal issues. Medicolegal issues that may arise from telemedicine include patients’ consent to disclose their health and genetic data, data security (data immortality and digital health footprints) and software and hardware errors.^17^ A lack of in-person physical examination and reliance on artificial intelligence interpretation can also yield cognitive bias and increase the risk of medical malpractice.^10,18^

The doctors were also neutral about telemedicine’s ability to save government healthcare costs in the present study. This finding reflects their lack of knowledge on the health economics of telemedicine, where although the initial set up cost of telemedicine (training and equipment) is high, the time and cost-effectiveness of its implementation is seen once the service is well established.^10^

The majority of the doctors in this study agreed that telemedicine can reduce workload, can be an alternative to physician consultation with patients and is time- and cost-effective for both patients and healthcare workers. Similarly, a prior study found that telemedicine can be safe and cost-effective.^18^ The MOH Malaysia implemented the Virtual Clinic programme in 2019 at five selected primary care clinics with the aim of increasing the feasibility of healthcare and reducing crowds at healthcare clinics. In the present study, most doctors agreed on the potential of ICT in healthcare and the necessity of telemedicine during a pandemic. With the emergence of the COVID-19 pandemic in 2019, countries worldwide have employed telemedicine in the healthcare field to provide medical services. The rapid uptake of telemedicine during the pandemic was attributed to the requirement for social distancing and reducing the risk of transmission.^19^

Similar to the findings by Albarrak et al.,^10^ the primary care doctors in this study had a positive perception towards telemedicine and perceived practising telemedicine as appropriate in the current setting. Interestingly, the doctors who had a working experience of ≤8 years had a significantly better perception towards telemedicine. These findings are comparable to the reports by Thong et al. and Gaggioli et al. that senior doctors are more reluctant to adopt telemedicine due to reluctance to accept changes in well-established clinical procedures and less familiarity with telemedicine technologies than their younger colleagues.^20,21^ In the present study, the doctors who had prior interaction with patients via emails, WhatsApp or Telegram also had a significantly greater perception towards telemedicine. This finding suggests that the doctors may have had positive experiences during prior usage of telemedicine modalities in their interaction with patients.

### Limitations

This study evaluated the perception of primary care doctors towards telemedicine in the city of Kuching, which covers urban and suburban areas. Therefore, the findings do not represent doctors in other parts of Sarawak, which has expansive rural areas. The study was also limited to doctors in government primary care settings and did not include those in hospitals or private sectors. Further, there is a possibility of response bias.

### Recommendations

According to Bashshur et al., successful implementation of telemedicine depends on three main pillars: improved access, enhanced quality and cost containment.^22^ Government initiative is needed in the adoption of telemedicine. This will allow standardisation of guidelines and wider access to telemedicine among the public.^23^ The benefits of a centralised system with government support have been shown during the COVID-19 pandemic through the initiation of online tracking applications (e.g. MySejahtera). There is benefit in the utilisation of a centralised system for telemedicine.

Telemedicine should also be financially viable for both healthcare providers and the public. This can be achieved through planning and clarification of policymakers on infrastructure, payment schemes or reimbursements. This has been observed in multiple countries (e.g. United Kingdom, Germany, Sweden, France and Australia) since the start of the COVID-19 pandemic.^24^ In these countries, governments and insurance companies have propagated the coverage of various teleconsultation methods tremendously.

Providing continuous training or workshops regarding telemedicine (guidelines, medical applications and technology) to all healthcare providers is suggested to increase the familiarity towards telemedicine. It is important to highlight possible medicolegal issues that may arise and ways to tackle them and to expose doctors to the health economics of telemedicine. Concurrently, ongoing technological support and educational initiatives for both clinicians and the public such as conferences, seminars and informative advertisements would be effective to increase awareness on telemedicine.

The development of telemedicine implementation guidelines that address primary care doctors’ perceptions especially during a period of pandemic is vital and can be applicable in future years.^25^

## Conclusion

The familiarity of primary care doctors at public healthcare clinics towards telemedicine is low. However, their perception is good in a majority of areas. Overall, primary care doctors agree on the appropriateness of telemedicine implementation. The positive uptake of telemedicine among primary care doctors is one of the key factors in developing telemedicine technology. With strategies focusing on key areas, including technological support and continuous education on telemedicine especially on medicolegal issues and telemedicine health economics, telemedicine in Malaysia has the potential to be propagated to greater heights.

## Data sharing statement

Data obtained and analysed are available from the corresponding author upon reasonable request.

**How does this paper make a difference in general practice?**

- This study found that the familiarity of primary care doctors towards telemedicine was still low. Thus, strategies to increase their familiarity must be planned.
- The study identified areas of good uptake that can encourage telemedicine usage.
- Areas of concern that could be a focus in the development and implementation of telemedicine by the government were identified.
- The study assessed the readiness of primary care doctors to implement telemedicine.
- The study also evaluated the types of cases that primary care doctors perceived as suitable for telemedicine consultations.
